# Supplementary material for: The interactome of intact mitochondria by cross-linking mass spectrometry provides evidence for coexisting respiratory supercomplexes
Source: Mol Cell Proteomics. 2017 Dec 8;17(2):216–32. doi: 10.1074/mcp.RA117.000470 (PMC5795388; doi:10.1074/mcp.RA117.000470)
Supplement: Supplemental Data [file supp_RA117.000470_133922_0_supp_23978_fzffwf.zip › spectra_annotation/mito_spectra_annotation/419-1-12-1-7-1.pdf]

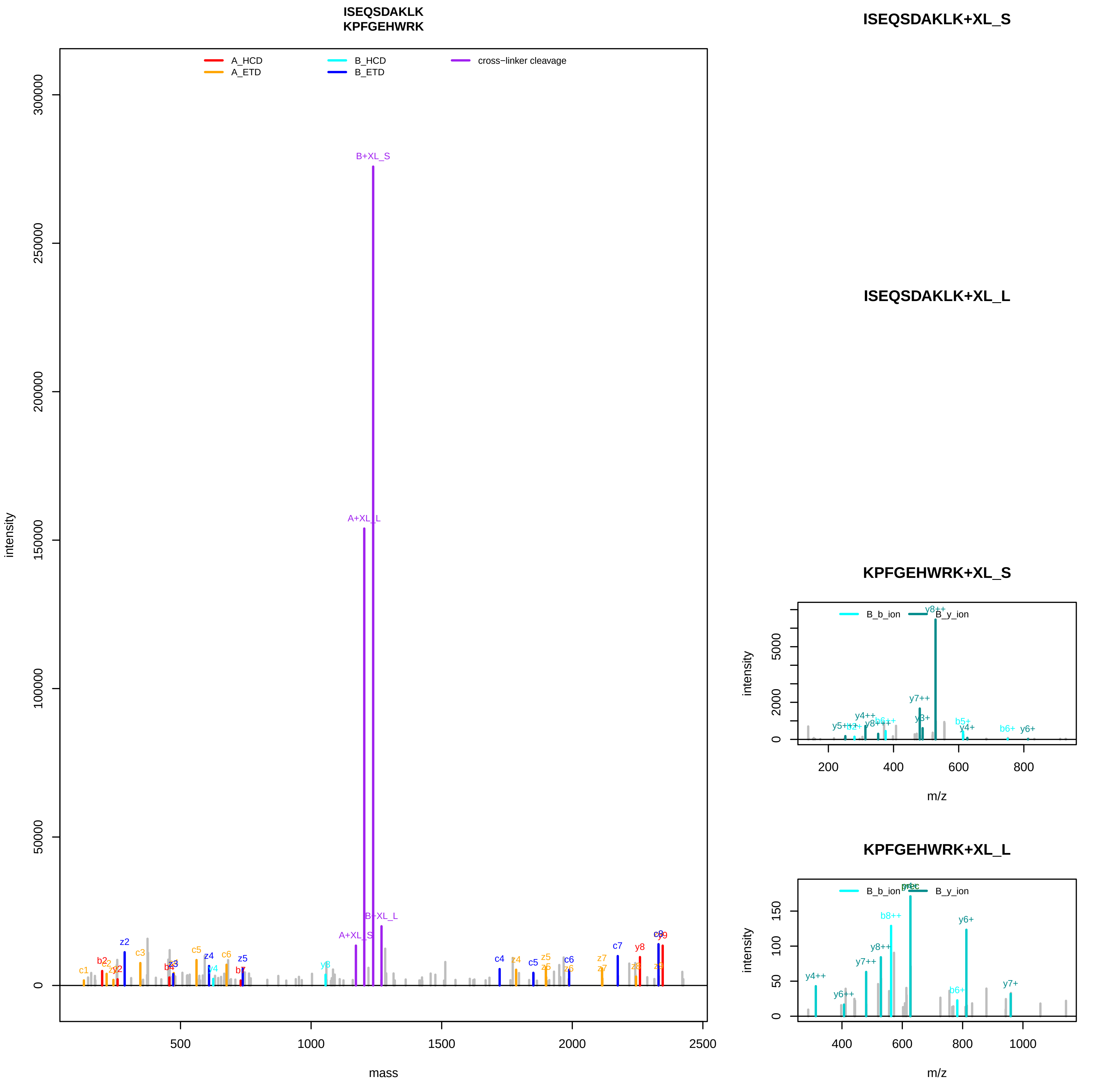
ISEQSDAKLK
KPFGEHWRK
A_HCD
A_ETD
B_HCD
B_ETD
cross−linker cleavage
B+XL_S
A+XL_L
B+XL_L
A+XL_S
intensity
mass
0
50000
100000
150000
200000
250000
300000
500
1000
1500
2000
2500
ISEQSDAKLK+XL_S
ISEQSDAKLK+XL_L
KPFGEHWRK+XL_S
B_b_ion
B_y_ion
y8++
y7++
y4++
y3+
b5+
y4+
b6+
y6+
m/z
0
2000
5000
200
400
600
800
KPFGEHWRK+XL_L
prec
b8++
y6+
y8++
y7++
y4++
y6++
b6+
y7+
0
50
100
150
400
600
800
1000
